# Supplementary material for: The lncRNA Statera regulates synaptogenesis by repressing the retrotransposon copia in Drosophila
Source: bioRxiv. 2026 Sep 5:2026.09.01.748680. Preprint. [Version 1] doi: 10.64898/2026.09.01.748680 (PMC13684589; doi:10.64898/2026.09.01.748680)
Supplement: Supplementary file 1 [file NIHPP2026.09.01.748680V1-supplement-1.pdf]

bioRxiv preprint doi: https://doi.org/10.64898/2026.09.01.748680; this version posted September 5, 2026. The copyright holder for this preprint (which was not certified by peer review) is the author/funder, who has granted bioRxiv a license to display the preprint in perpetuity. It is made available under a CC-BY 4.0 International license.

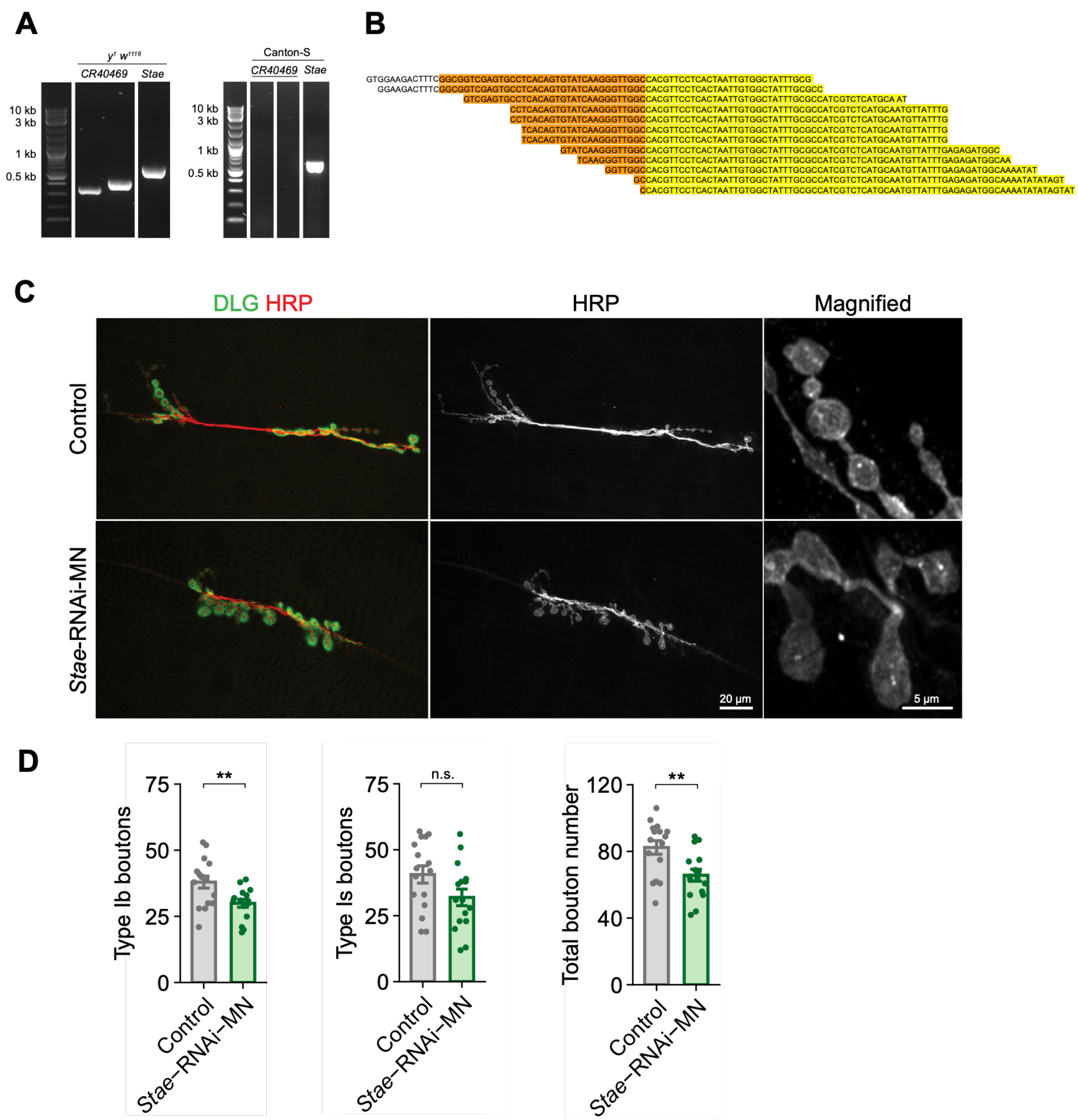

Figure S1. Motor neuron-specific *Stae* RNAi causes altered NMJ morphology. (A) DNA gel images show that *Stae* is present in both wild-type CS and y^1 w^{1118} flies from BestGene, while the *CR40469* homologous sequence is not detected in the lab CS strain. **(B)** Reads from whole-genome sequencing show that *Stae* fragments, but not *CR40469* fragments, are detected in the lab CS strain. Sequences matching the annotated 5' region of *CR40469* are highlighted in yellow. Sequences upstream of the annotated *CR40469* sequence are highlighted in orange. These upstream sequences match the 5' region of *Statera*, but not the genomic sequence upstream of *CR40469*. **(C)** Fluorescence images of NMJ preparations from motor neuron-specific *Stae* RNAi animals. **(D)** Quantification of NMJ morphology in motor neuron-specific

bioRxiv preprint doi: https://doi.org/10.64898/2026.09.01.748680; this version posted September 5, 2026. The copyright holder for this preprint (which was not certified by peer review) is the author/funder, who has granted bioRxiv a license to display the preprint in perpetuity. It is made available under a CC-BY 4.0 International license.

Stae RNAi. DLG, Discs large (postsynaptic marker); HRP, horseradish peroxidase (presynaptic marker). Data are presented as mean ± SEM, with each dot representing a biological replicate. Statistical analyses were conducted using Student's t-test. ns, not significant; * $p<0.05$; ** $p<0.01$; *** $p<0.001$.

bioRxiv preprint doi: https://doi.org/10.64898/2026.09.01.748680; this version posted September 5, 2026. The copyright holder for this preprint (which was not certified by peer review) is the author/funder, who has granted bioRxiv a license to display the preprint in perpetuity. It is made available under a CC-BY 4.0 International license.

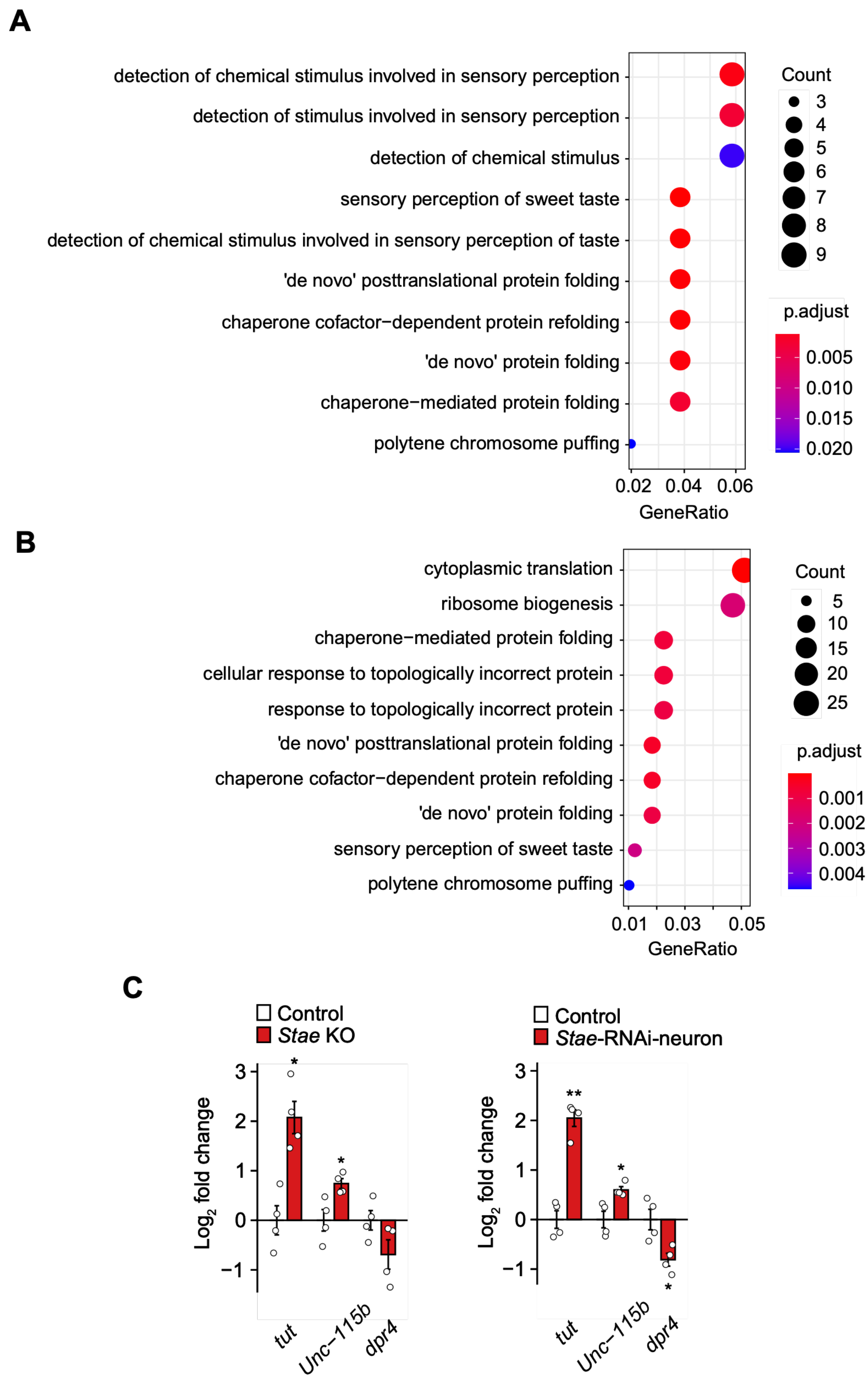

bioRxiv preprint doi: https://doi.org/10.64898/2026.09.01.748680; this version posted September 5, 2026. The copyright holder for this preprint (which was not certified by peer review) is the author/funder, who has granted bioRxiv a license to display the preprint in perpetuity. It is made available under a CC-BY 4.0 International license.

Figure S2. Biological processes associated with *Stae*. (A and B) GO analysis of differentially expressed genes in **(A)** neuron-specific *Stae* RNAi and **(B)** muscle-specific *Stae* RNAi. The analyses identified common biological processes related to protein folding, as well as tissue-specific processes such as sensory perception in the CNS and cytoplasmic translation in BWMs. **(C)** dPCR validation of candidate genes downstream of *Stae* in *Stae* KO and RNAi larvae. Total RNA was extracted from the CNS, and the candidate RNA levels were determined using target-specific primer sets and normalized to *rpl32* levels. Data are presented as mean ± SEM, with each dot representing a biological replicate. Statistical analyses were conducted using Student's t-test. ns, not significant; * $p<0.05$; ** $p<0.01$; *** $p<0.001$.
